# A replication-competent deltavirus from the marsupial fat-tailed dunnart Sminthopsis crassicaudata

**DOI:** 10.1099/jgv.0.002203

**Published:** 2026-01-16

**Authors:** Zoé Denis, Valérie Courgnaud, Marcos de la Peña, Karim Majzoub

**Affiliations:** 1 Institut de Génétique Moléculaire de Montpellier (IGMM), Univ Montpellier, CNRS, Montpellier, France; 2 Instituto de Biología Molecular y Celular de Plantas, Universidad Politécnica de Valencia-CSIC, Valencia, Spain

**Keywords:** deltavirus, Kolmioviridae, marsupial virus, metatranscriptomics, virus discovery

## Abstract

Deltaviruses are circular, negative-sense RNA agents that replicate autonomously but depend on heterologous envelope glycoproteins for spread. Only partial sequences of deltaviruses had been reported from marsupials. By reanalysing public metatranscriptomes from the Australian fat-tailed dunnart (*Sminthopsis crassicaudata*), we assemble the first complete marsupial deltavirus genome and test its replication in human and animal cells. The fat-tailed dunnart deltavirus (FtDDeV) is a 1,680-nt circular RNA that folds into a canonical unbranched rod-like structure and encodes a 195-aa delta antigen (FtDDAg). Genomic and antigenomic HDV-like ribozymes are present and conserve catalytic core motifs. Phylogenetic analyses cluster FtDDAg with the Tasmanian devil sequence, and both are quite close to RDAg from the neotropical rodent species *Proechimys semispinosus*. A dimeric FtDDeV cDNA replicon supports time-dependent DAg accumulation in human, simian, rodent and Tasmanian devil cells, with faster kinetics in rodents and marsupial cells. FtDDAg accumulation patterns in host nuclei show characteristic viral hubs, observed with other deltaviruses. No obvious coinfecting helper viruses were detected in FtDDeV-positive libraries. Our study extends the confirmed host range of deltaviruses to marsupials and provides a replication-competent clone to investigate helper usage, host restriction and deltavirus evolution.

## Introduction

The *Kolmioviridae* family (referred to here as deltaviruses) includes viruses with circular, single-stranded, negative-sense RNA genomes of ~1.7 kb in length [1]. This family of viruses was recently named after the Finnish word kolmio (triangle), referring to the Greek letter Δ, which is related to the name of the first genus identified: deltavirus, which includes the hepatitis D virus (HDV). Indeed, the main and best-characterized member of this family is HDV, first identified in 1977 in human patients suffering from severe hepatitis and co-infected with hepatitis B virus (HBV) [2,3]. HDV was initially thought to be strictly associated with human infections and hypothesized to either arise from the human transcriptome [4] or from plant viroids [5], which are single-stranded circular RNAs that infect plants. However, recent discoveries indicate that HDV has a rather long evolutionary history. Over the past few years, HDV-like sequences have been discovered in a wide range of animal species, revealing that deltaviruses are far more diverse and widespread than previously thought, with 11 genera known to infect a broad spectrum of animals. Indeed, RNA sequencing datasets from a variety of animals have uncovered HDV-like elements in several mammalian species, including bats, woodchucks, deer and rodents [6,8] but also in birds [7,9, 10], reptiles [11], amphibians, fish and insects [12]. These findings revealed a greater diversity and complexity within the deltaviruses, showing their large host range and strongly suggesting a zoonotic origin of HDV [6].

As the prototypical representative of deltaviruses, HDV displays key hallmarks of this viral family, including a negative-sense RNA genome with a rod-like structure conferred by its high self-complementarity [13,14]. It possesses a minimal genome containing both genomic and antigenomic ribozymes, which are crucial for viral replication, and a single ORF encoding two forms of a protein, the small and large hepatitis delta antigen (S- and L-HDAg) [13,14]. The constrained coding capacity of the HDV genome makes this virus entirely reliant on host cellular machinery, particularly RNA polymerase II [15,16] (but other cellular polymerases, such as RNA polymerase I or III, have also been suggested [17] which is required for replication and transcription, as well as other host factors that have yet to be identified.

Importantly, deltaviruses cannot produce their own envelope glycoproteins, making them non-autonomous viral entities. Instead, they rely on hijacking heterologous envelope glycoproteins from co-infecting helper viruses to enable their assembly, spread and entry into naïve cells. To date, HBV is the only known helper virus associated with HDV, providing HBV surface antigen to form HDV’s viral envelope. Hence, this envelope dictates its liver tropism, since (Na(+)- Taurocholate Co-transporting Polypeptide) NTCP (Sodium Taurocholate Co-transporting Polypeptide), the HBV entry receptor, is mainly expressed in the liver [3,18]. In fact, deltavirus host tropism is thought to be principally determined by the tropism of their helper viruses. However, for most animal-associated deltaviruses, helper viruses remain largely unknown. Currently, insights into potential helper viruses rely primarily on RNA-seq evidence of viral co-infections in deltavirus-positive hosts. Notably, no co-infections with *Hepadnaviridae*-related viruses have been co-detected in animals where new deltaviruses were found [6,9,11, 12]. Nonetheless, experimental evidence suggests that HDV can form infectious particles by utilizing envelope glycoproteins from other viral families, such as *Flaviviridae* and *Rhabdoviridae* [19]. Among animal-associated deltaviruses, envelope glycoproteins from members of the *Arenaviridae* family have been shown to enable the formation of *Boa constrictor* snake deltavirus (SDeV) infectious particles [20,21], while the rodent deltavirus (RDeV) from *Proechimys semispinosus* has so far only been shown to be infectious when packaged with *rhabdovirus* glycoproteins [20]. Interestingly, co-infections with avian bornaviruses have recently been detected in most RNA-seq datasets positive for *Serinus canaria*-associated deltavirus [22].

Our recent observations on the potential of various deltavirus members (HDV, RDeV and SDeV) to replicate in different animal species and cell types [20], combined with suggested host-shifting events during deltavirus evolution [6], raise important questions about their capacity to cross the species barrier and their potential for rapid adaptation to new and diverse animal hosts. The identification of new HDV-like agents in species where deltaviruses have not previously been described could offer powerful insights into the origin and evolution of these satellite viruses. Such findings would also deepen our understanding of how these entities spread and adapt to different animal hosts. Moreover, access to the viromes of infected individuals is crucial for uncovering mechanisms that govern the packaging and transmission of these satellite viruses in nature.

The recent study carried out by Harvey *et al*. [23] provided new insights into the virome of Dasyuromorphia, a marsupial order containing most of the carnivorous marsupials found exclusively in Australia [23]. Through RNA-seq analysis, the authors identified two novel partial HDV-like antigen sequences potentially belonging to marsupial deltaviruses, detected in a fat-tailed dunnart and a Tasmanian devil. In this paper, we identify and characterize the first complete sequence of a marsupial deltavirus*,* from the Australian fat-tailed dunnart (*Sminthopsis crassicaudata*) that we call fat-tailed dunnart deltavirus (FtDDeV).

## Methods

### Bioinformatic analysis

RNA sequencing datasets from the Sequence Read Archive (SRA), comprising a total of 40 libraries derived from *S. crassicaudata* tissues across BioProjects PRJNA1028148, PRJNA676111, PRJNA356957 and PRJNA399240, were screened through blast [24] for the presence of reads corresponding to deltavirus sequence fragments [23]. BioProject PRJNA1028148 yielded five libraries with viral reads, which underwent *de novo* assembly using the RNAviralSpades workflow from the SPAdes genome assembler version 4.1.0 [25]. Assembled contigs were used to search for the presence of delta antigen ORFs (blastp) and deltavirus ribozymes (INFERNAL 1.1.5, September 2023 release). Secondary structure predictions and minimum free-energy folding calculations for the assembled viral genomes were generated using the RNAfold [26] and mFold web servers [27]. Sequences of the delta antigens were aligned with muscle 5.3 [28] with default parameters. Predicted structure of the delta antigens was done with AlphaFold 3 server [29].

### Detection of co-infected viruses

All previous libraries containing deltavirus sequences were analysed for RNA and DNA viruses by examining with Palmscan [30], which detects RNA-dependent RNA polymerase fingerprints, and VirFinder [31], which identifies viral sequences based on sequence features.

### Phylogenetic analysis

The phylogenetic tree was built using the maximum likelihood method implemented in IQ-TREE 2.4.0 [32]. The best-fit evolutionary model according to the Bayesian information criterion was the JTT+F+G4 substitution model. The final tree was obtained after iterative optimization of candidate trees resulting from parsimony and neighbour-joining methods to best fit the sequence data, considering evolutionary rate variation. Branch supports were assessed using 1,000 replicates of the ultrafast bootstrap approximation and the SH-like approximate likelihood ratio test.

### Cell lines

HEK-293T, Huh7.5, CHO, Vero and Tasmanian devil fibroblast cell lines were cultivated in Dulbecco Modified Eagle Medium supplemented with 10% FBS, non-essential amino acids and 100 U penicillin ml^−1^, 0.^1^ mg streptomycin ml^−1^ at 37 °C and 5% CO2-air atmosphere.

### Cloning of the fat-tailed dunnart deltavirus genome dimer

Full-length sequence corresponding to the genome of FtDDeV was synthesized as a gBlock Gene Fragment (Integrated DNA Technologies). The FtDDeV was then amplified by PCR and cloned in genomic orientation into the EcoRV and XbaI sites of the pcDNA3 expression vector. The resulting construct (pcDNA3/FtDDeV) was then amplified by PCR along with the FtDDeV genome, and both PCR products were ligated together using T4 DNA ligase (New England Biolabs), yielding an expression plasmid containing a dimer of the FtDDeV genome (pcDNA3/2XFtDDEV).

### Immunoblotting

HEK-293T, Huh7.5, CHO, Vero and Tasmanian devil fibroblast cells were transfected with pcDNA3/2XmmDeV (marmota monax deltavirus) [7] or pCDNA3.1/2XFtDDeV plasmids using the jetPEI^®^ DNA Transfection Kit (Polyplus #101000020) according to the manufacturer’s instructions. Transfected HEK-293T, Huh7.5, CHO, Vero and Tasmanian devil fibroblast cells were harvested at 3, 6 and 9 days after transfection and then lysed in 1X RIPA buffer (Merck #20–188) supplemented with a protease inhibitor cocktail (Thermo Scientific #87785) and analysed by immunoblotting using polyclonal antibodies targeting hepatitis delta antigen (HDAg) purified from serum of an HBV/HDV coinfected patient as previously described [20]. Proteins were separated on a 4–20% Mini-PROTEAN TGX gel (Bio-Rad #4561093) and transferred onto PVDF membrane using a Trans-Blot Turbo Transfer System (Bio-Rad #1704150EDU) and probed. Loading was controlled by probing with *β*-actin. *β*-Actin detection was performed using an anti-*β*-actin monoclonal antibody (1:1000; MA5-11869, Invitrogen). iRDye labelled specific secondary antibodies were detected using the Odyssey M Infrared Imaging System (LI-COR Biosciences #3350).

### Immunofluorescence staining

Experiments were performed using DFT or Huh7.5 cells transfected with 1 μg of either mmDeV or FtDDeV plasmids using the jetPEI^®^ DNA Transfection Kit according to the manufacturer’s instructions.

For DFT cell experiments, at 5 days post-transfection, cells were washed with PBS (Eurobio #CS1PBS01-01) and then fixed and permeabilized for 20 min at RT (Room temperature) in PBS containing 4% paraformaldehyde (Electron Microscopy Sciences #15714) and 0.2% Triton X-100 (Bio-Rad #1610407). Blocking was performed in PBS, with 0.5% BSA (Sigma-Aldrich #A3059) for 30 min at RT, followed by 2 h of incubation with polyclonal antibodies targeting Human DAg [20], diluted 1:500 in the same blocking buffer. After three washes with PBS, cells were incubated for 45 min at RT with Alexa Fluor 488-conjugated goat anti-human IgG secondary antibody (Invitrogen, #A11013) diluted 1:500 and 300 nM DAPI (Invitrogen #D21490) in PBS. Cells were washed and imaged using an ImageXpress^®^ Pico Automated Cell Imaging System (Molecular Devices) with a 10× lens.

For confocal microscopy, transfected Huh7.5 cells were grown on microscope cover glasses (Marienfeld #0102052) in 6-well plates and fixed at 5 days post-transfection with PBS containing 4% paraformaldehyde (Electron Microscopy Sciences #15714) for 20 min at RT. Cells were then permeabilized for 20 min at RT in PBS containing 0.2% Triton X-100 (Bio-Rad #16104067). Blocking was performed in PBS, 0.2% Triton X-100, 2% BSA for 45 min at RT, before overnight incubation at 4 °C with polyclonal antibodies targeting Human DAg [20], diluted 1:500 in the same blocking buffer. Cells were washed three times in PBS and incubated with Alexa Fluor 488-conjugated goat anti-human IgG secondary antibody (Invitrogen, #A11013) diluted 1:500 and 300 nM DAPI (Invitrogen #D21490) in PBS for 2 h in the dark. Cover glasses were then washed with PBS and mounted in ProLong Gold antifade reagent (Invitrogen #P36930).

Images were acquired using a Zeiss LSM980 confocal microscope (controlled with Zen blue 3.7) on an Airyscan 2 detector in Super Resolution mode with a 40× oil objective 1.3NA.

## Results

### A full deltavirus genome in the marsupial *S. crassicaudata*

Recent discoveries of novel deltaviruses in diverse mammals, but also in other non-mammalian vertebrates and even invertebrate animals [6,9,11, 12, 22], have revitalized the field of infectious subviral circular RNA agents. The recent report of putative genomic fragments of deltaviruses in two marsupials may allow us to build the evolutionary history of these agents among mammals, including humans. Harvey and collaborators [23] reported a partial sequence for a putative FtDDeV genome in eye tissues of the Australian fat-tailed dunnart (run SRR9673767, BioProject PRJNA554238) [23]. Our bioinformatic analysis, ribozyme and delta antigen searches (see the ‘Methods’ section) revealed the presence of FtDDeV sequences in four other RNA-seq datasets obtained from diverse Australian fat-tailed dunnart tissues (Table S1, available in the online Supplementary Material). A full circular genome of 1,680 nt was assembled from one dataset (SRR26386756 run, endometrium tissue), whereas partial genomic sequences were detected in three other runs from the same BioProject, two of them from two replicates (SRR26386756 and SRR26386778) from the same biological sample (Table S1).

**Fig. 1.**
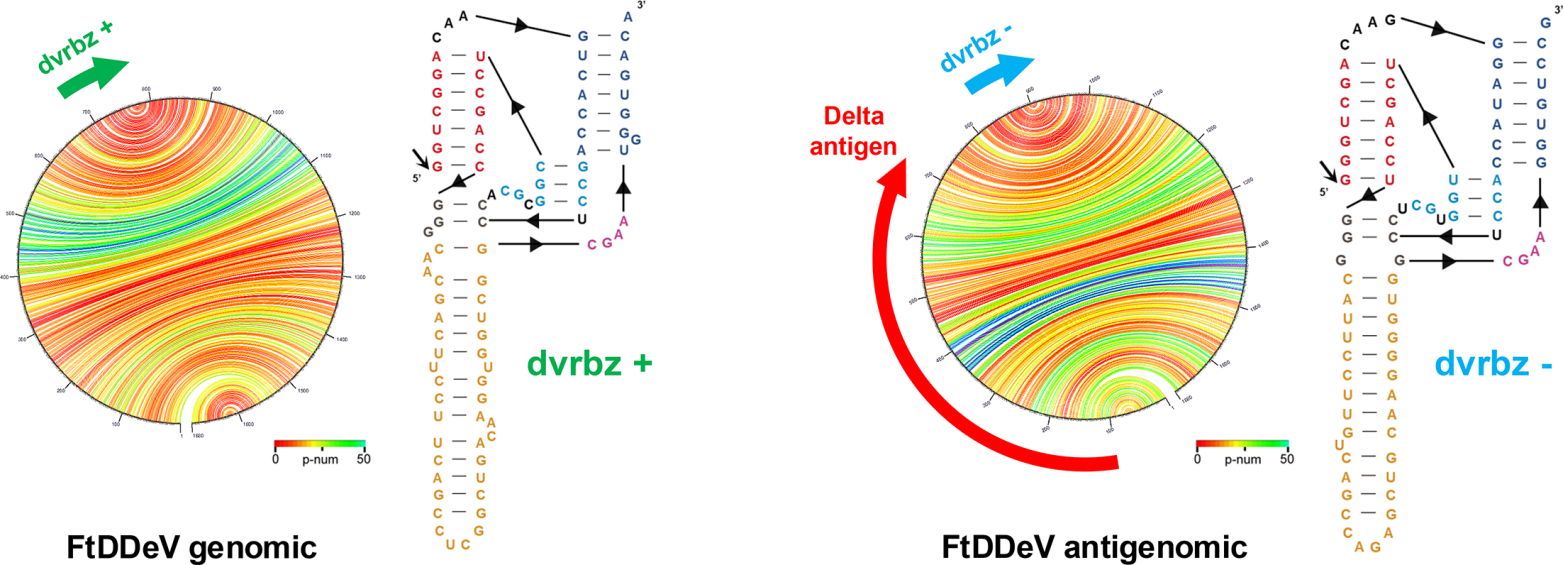
Secondary structure for the FtDDeV and their self-cleaving ribozymes corresponding to the genomic (left) and antigenomic (right) strands of the virus. Characteristic rod-like folding is shown with Jupiter plots, where each line shows the predicted base-pairing within the RNA genome, coloured by base-pairing confidence score (p-num). Small arrows indicate the position of the deltavirus ribozymes (dvrbz). The red arrow indicates the position of the delta antigen ORF encoded by the antigenomic FtDDeV sequence.

The full FtDDeV circular 1,680 nt RNA genome shows a GC content of 54.58% and adopts the classical rod-like secondary structure in both genomic and antigenomic RNA polarities (Fig. 1). This composition sits between the GC-rich mammalian genomes of HDV (~60%) and RDeV (62.9%) [8,33] and the lower-GC clades infecting reptiles (53.3%), birds (51%) and fish (46.3%) [9,11, 12]. Minimum-free-energy folding of the circular RNA genome produces the canonical unbranched rod in which 1,143/1,680 nt (68.0%) are base-paired (Fis 1 and S1A). Pairing coverage is slightly below that of HDV (~74%) and the RDeV (~71%) but above those reported for SDeV (~64%) and avian deltaviruses (~60%), mirroring the GC content gradient across the family.

 The presence of ambisense deltavirus ribozymes can also be detected in both polarities, whose primary sequences resemble an intermediary sequence of the mammalian and sauropsid deltavirus ribozymes [34] (Figs 1 and S2). FtDDeV genome harbours canonical genomic and antigenomic HDV-like ribozymes; their catalytic cores are sequence-identical to those of prototype HDV (Fig. 1) and retain all residues required for self-cleavage [35]. A single antigenomic ORF encodes a 195-aa protein termed FtDDAg (22.2 kDa, pI ≈ 10.0, net charge +9.6). Length, charge and the Lys/Arg-rich N-terminal RNA-binding motif match those of mammalian DAgs (195–196 aa; pI 9.8–10.4), whereas reptile and avian DAgs are marginally longer (199–204 aa) and slightly less basic (pI ≈ 9.4–9.6) [6,8, 9, 11, 12, 33]. Interestingly, the FtDDAg ORF does not end with an amber stop codon UAG like its HDAg counterpart, but rather an ochre UAA stop codon. A potential A-to-I ADAR1 editing of FtDDeV antigenome would change the ochre UAA codon to a UGA, which would still be a stop codon. Moreover, the flanking RNA lacks the characteristic 4-nt asymmetric bulge and an A-C mismatch at the potentially edited A nucleotide, both crucial for efficient editing [36,39] and known to promote ADAR1 editing at the amber/W site seen in HDV (UAG→UGG) [40]. These two properties in the FtDDeV genome make it unlikely that the production of a large DAg by this virus through an editing mechanism similar to that observed in HDV [38] (Fig. S1, Supplementary Material 1). Importantly, apart from the HDV, no large DAg has been observed to date in animal deltaviruses [1,8, 11, 12, 20].

### No other RNA or DNA viruses were detected in the FtDDeV-containing datasets

Bioinformatic analysis through Palm domains/Palm annotation/Serratus [30] pipelines (see the ‘Methods’ section) searching for RNA viruses was carried out but did not reveal the presence of any RNA virus genome, other than FtDDeV, in any of the five *S. crassicaudata* datasets. Regarding DNA viruses, whereas some viral genomes were detected through STAT analysis [41], the few putative reads resembling viral sequences occur at very low levels (below 0.01%) and correspond mostly to human viruses (such as herpes or papillomaviruses). Using VirFinder software [31], up to 179 contigs with putative viral origin (score >0.99 with a length >300 bp) were detected in the FtDDeV containing runs. However, blast searches confirmed that 140 contigs likely corresponded to genomic sequences from either marsupials or placental mammals, whereas 39 did not find significant similarity with any known sequence. Of note, FtDDeV sequences were actually not detected by VirFinder.

### FtDDAg places marsupial deltaviruses close to their placental mammal counterparts

**Fig. 2.**
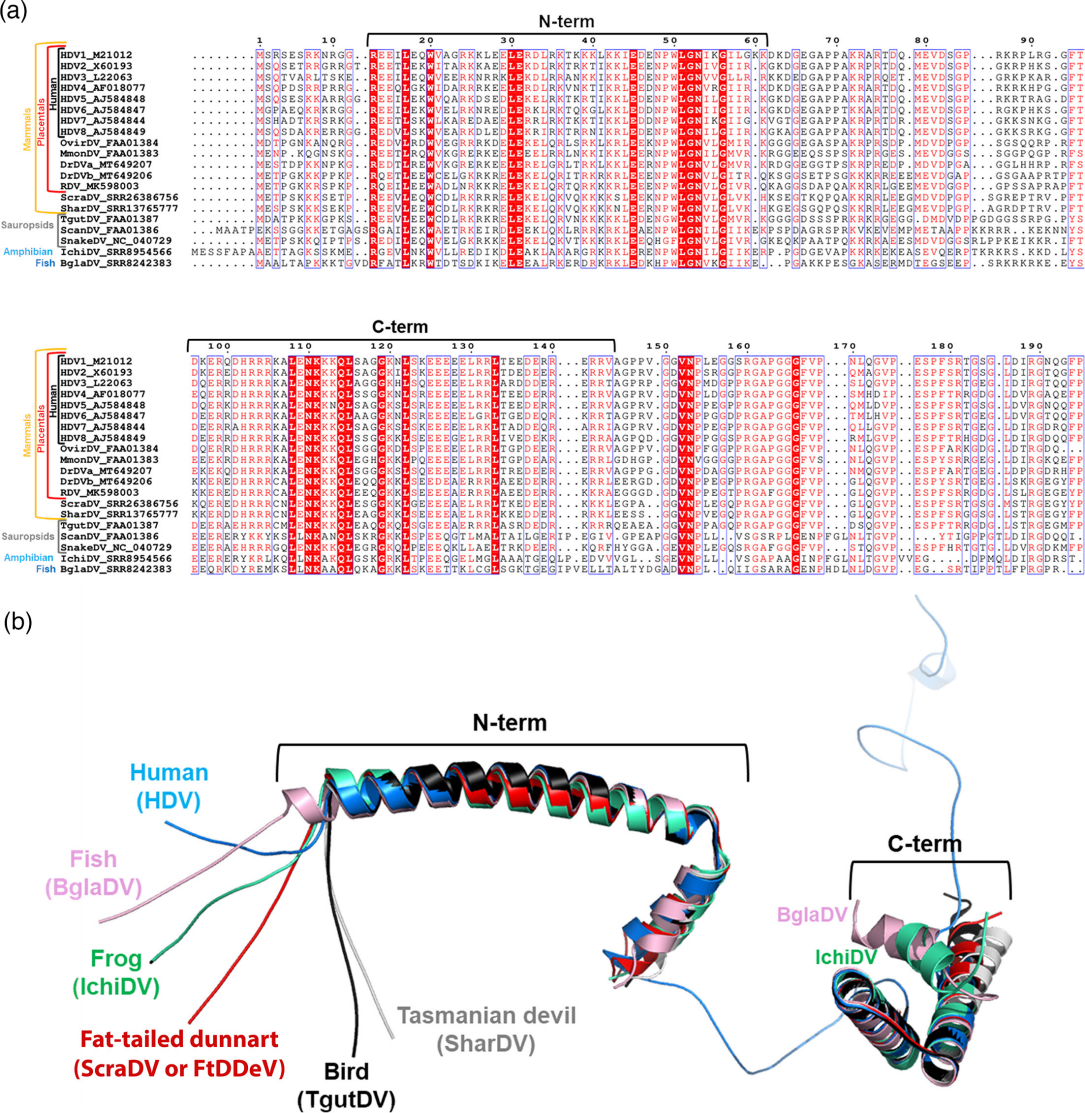
(**a**) Sequence alignment of delta antigen sequences of vertebrate deltaviruses. The two structured domains at the N- and C-terminus are indicated with brackets. (**b**) Predicted 3D structure of the whole delta antigen encoded by the human HDV8 (in blue). The conserved N- and C-terminus domains of the delta antigens from five vertebrate deltaviruses were superposed to the human one. While both structured domains of delta antigens in marsupials and birds adopt folds closely resembling those of human and other mammalian deltaviruses, the C-terminal domain of the delta antigen in fish and amphibians exhibits a divergent conformation, with one of the two predicted helices adopting a distinct fold.

**Fig. 3.**
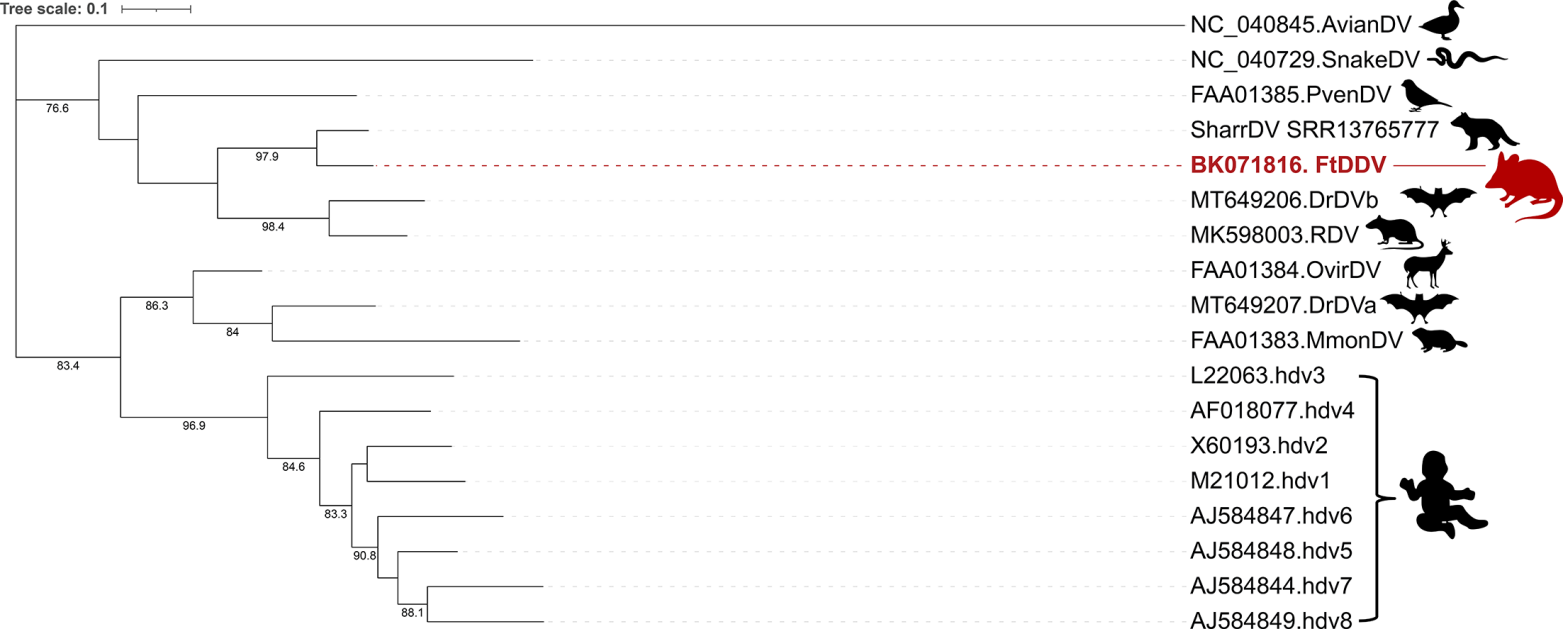
Phylogenetic tree of vertebrate deltaviruses based on delta antigen protein sequences. The phylogeny was estimated using the JTT+F+G4 substitution model. Branch support >70% is indicated. The scale bar indicates the number of amino acid substitutions per site. Animal silhouettes indicate the host species.

The sequence of the delta antigens among mammals is highly divergent [6]. The alignment of delta antigen (DAg) encoded by known deltaviruses from diverse vertebrates, including placentals, marsupials, reptiles, birds and fish (Fig. 2a), shows that two regions of the DAg are conserved among all of them: the coiled-coil motif at the N-terminus and the helix-loop-helix closer to the C-terminus of the DAg, which are both connected by a highly variable region with a predicted unstructured conformation. Whereas the predicted coiled-coil structure is identical for all deltaviruses, including FtDDeV, the predicted helix-loop-helix (the second helix in fact) is slightly different for fish (Fig. 2b). Phylogenetic analysis groups the two marsupial DAgs, which form a clade with the rodent and one bat deltavirus antigen. Interestingly, this clade seems to be closer to the snake or bird DAgs than to other mammalian DAgs, including the human ones (Fig. 3).

### The fat-tailed dunnart deltavirus cDNA clone replicates in human and animal cells

**Fig. 4.**
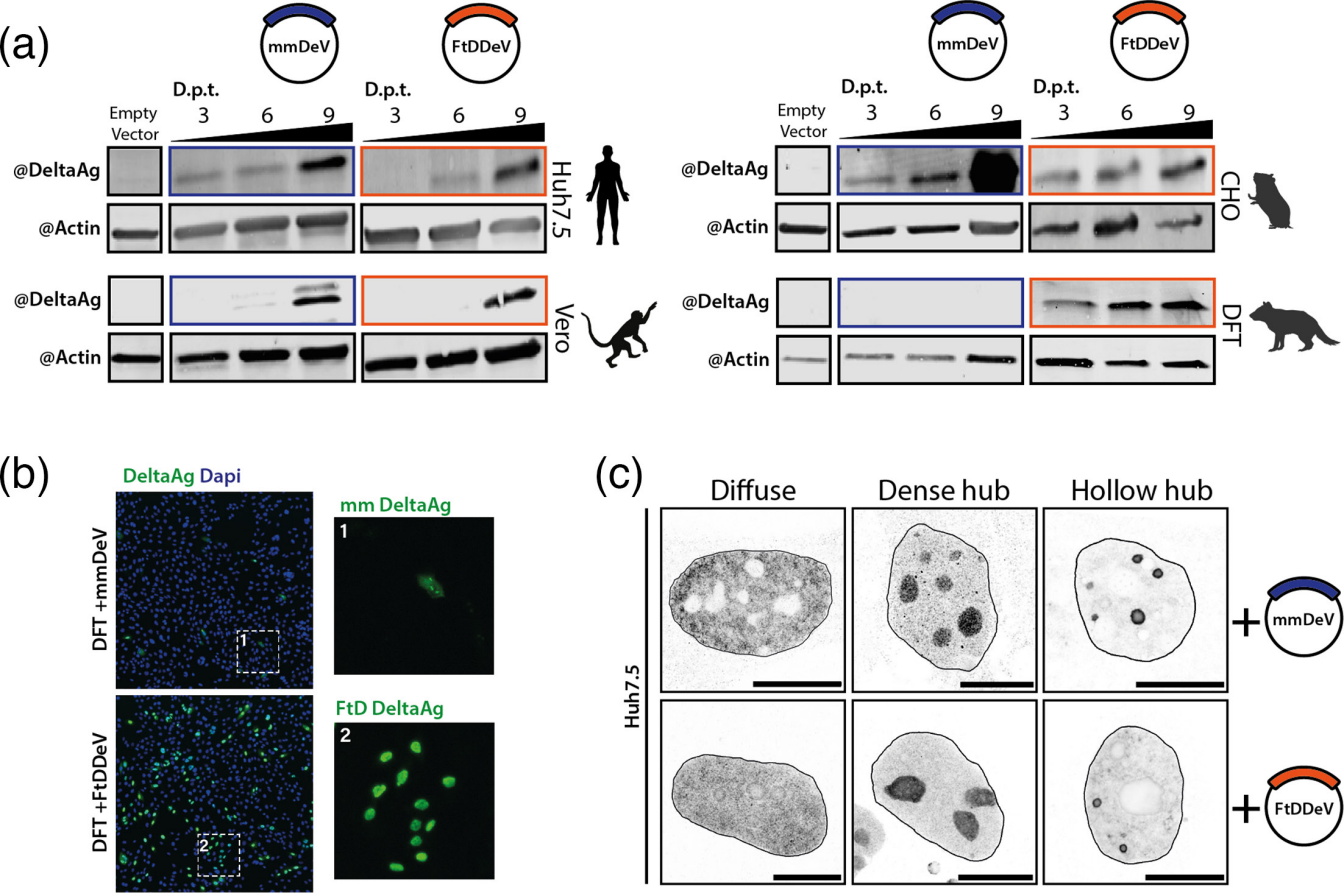
mmDeV and FtDDeV antigen accumulation and subcellular localization in mammalian cell lines. (**a**) Western blotting analysis of Huh7.5, Vero, CHO and DFT cells transfected with a mmDeV or a FtDDeV dimer-genome replicon. The delta antigen expression was assessed at 3, 6 and 9 days post-transfection (D.p.t) by Western blotting. *β*-Actin serves as a loading control. (**b, c**) Immunofluorescence detection of mmDeV and FtDDeV delta antigens in FTD (**b**) or Huh7.5 cells (**c**) after 5 days of transfection with mmDeV or FtDDeV dimer-genome replicons. Delta antigens were stained in green (**b**) or grey (**c**) by using polyclonal antibodies targeting the Human delta antigen and nuclei were stained in blue with DAPI (**b**) or delimited with a hatched line (**c**). Scale bars 10 µm.

As shown in previous studies, deltaviruses are able to replicate in a variety of tissues and animal species, including in cells belonging to hosts that differ from their natural reservoir [20,42]. To investigate the replication capacity of FtDDeV, we cloned the FtDDeV genomic sequence (GenBank accession number BK071816) as a dimer as previously described [8], in a pcDNA3 plasmid where its expression was driven by a CMV promoter [8,20]. We transfected the obtained FtDDeV replicon and a previously described marmota monax deltavirus (mmDeV) cloned similarly [7], as a control, in four cell lines derived from different species (Fig. 4a). These included the human liver-derived Huh7.5 hepatoma cell line, African green monkey Vero cell line, the Chinese hamster ovary CHO cell line and a marsupial fibroblast cell line (DFT) derived from the Tasmanian devil *Sarcophilus harrisii* (Fig. 4a). We used mmDeV as a control because RDeV, the closest known relative to FtDDeV, was shown to replicate efficiently and with rapid kinetics in a wide range of species [20], making it less suitable for studying host-specific replication features. Therefore, we selected mmDeV to serve as a more appropriate control for replication studies.

Cells were collected and lysed 3, 6 and 9 days post-transfection, and lysates were subjected to Western blot analysis for DAg detection, using cross-reactive polyclonal antibodies isolated from HDV-positive patients [20]. The FtDDAg was detected in all tested cell lines, with a molecular weight of about 22 KDa, in line with its theoretical size (Fig. 4a). Importantly, FtDDAg level increases over time, indicating that FtDDeV can replicate autonomously in all tested cells (Fig. 4a). Expectedly, no larger DAg band was detected at higher molecular weights in FtDDeV samples (Fig. 4a), even at later time points, suggesting that FtDDeV is different from HDV and only produces a small DAg species, similar to RDeV or mmDeV [7,20]. Interestingly, FtDDeV replication kinetics seemed to differ between cell lines. Indeed, FtDDeV replication in human (Huh7.5) and simian (Vero) cell lines was relatively slow, mirroring that of mmDeV, with a detectable DAg band appearing at 6- and 9 days post-transfection (Fig. 4a). In contrast, FtDDeV showed faster replication kinetics in hamster CHO cells and Tasmanian devil cells, with a FtDDAg visible as early as 3 days post-transfection (Fig. 4a). Surprisingly, while the marsupial DFT cell line supported FtDDeV replication the best (Fig. 4a), it was completely non-permissive to mmDeV replication, suggesting that this virus might be restricted in this cell line (Fig. 4a). Indeed, immunofluorescence analysis of DAg in DFT cells showed a much more pronounced accumulation of FtDDAg in nuclei of transfected cells (with 12.9% of FtDDAg positive cells) compared to marmota monax delta antigen (mmDAg) (with only 1.4% of mmDAg-positive cells) (Fig. 4b). This suggests that FtDDeV is better adapted to marsupial cell lines than mmDeV. A closer look at FtDDeV replicon-transfected Huh7.5 nuclei using confocal microscopy revealed characteristic deltavirus accumulation patterns (Fig. 4c). Indeed, three main hallmarks of HDV, RDeV and SDeV accumulation have previously been observed in infected cells: a diffuse DAg pattern, a dense hub and a hollow hub [20]. These three patterns could also be observed in FtDDeV-positive cells (Fig. 4c), indicating that this virus shares nuclear accumulation patterns and mechanisms with other deltaviruses.

## Discussion

Until recently, only a partial sequence of a deltavirus from the Australian fat-tailed dunnart, comprising a part of the DAg mRNA, had been described [23]. In this paper, using available metatranscriptomic datasets, we have identified and characterized the first complete genome sequence of a new deltavirus from marsupials (the Australian fat-tailed dunnart), which we term FtDDeV. Our present work, together with previous evidence of deltavirus sequence fragments identified from Tasmanian devils and other fat-tailed dunnart datasets [23], extends the known host range of the deltavirus family to a new taxonomic group (non-placental mammals): marsupials. FtDDeV presents all canonical features found in other deltaviruses, such as a negative-stranded circular genome, a self-cleaving ribozyme in both genomic and antigenomic strands and a single ORF encoding a protein with high similarity to DAgs found in other deltaviruses. Importantly, analysis of the FtDDAg amino acid content identified the mammalian RDAg found in spiny rats as the closest deltavirus relative, sharing ~70% of amino acid identity, and placed FtDDeV phylogenetically between placental mammals and sauropsids (Figs 2a and 3). The close phylogenetic relationship between FtDDeV and RDeV suggests that deltaviruses infecting marsupials share a common ancestor with those infecting placental mammals such as rodents and bats, but also with the deltavirus found in birds. This underlines once again that deltavirus phylogeny does not correspond to host phylogeny [6]. Interestingly, the close phylogenetic relationship between FtDDAg and Tasmanian devil DAg (Fig. 3) could indicate that an ancestor of deltavirus infected these species prior to their diversification or that deltaviruses underwent cross-species transmission or host-shifting between these two animal species in the past [6].

Our molecular characterization of FtDDeV replication demonstrates for the first time that FtDDeV can replicate autonomously in a marsupial cell line as well as in various cell lines from placental mammals, including human, simian and rodent cells (Fig. 4a). Interestingly, FtDDeV showed robust replication in CHO rodent cells, phylogenetically closer to marsupials than the more distant human or simian species, where FtDDeV replication kinetics were clearly slower (Fig. 4a). In contrast, the mmDeV, found in the groundhog (*Marmota monax*), a placental mammal, failed to replicate in marsupial DFT cells (Fig. 4a and b), highlighting either potential host-specific restriction or the lack of essential pro-viral host factors required for viral replication. This observation supports a hypothesis positing that FtDDeV has evolved in the presence of marsupial cellular machineries and has better adapted to marsupial hosts over time. Previous studies have already reported the ability of certain deltaviruses to replicate in organisms other than their original hosts [7,8, 20, 21]. Here, the capacity of FtDDeV to replicate in several mammalian hosts, placental or non-placental, despite its evolutionary divergence, highlights once again the wide host range of deltaviruses and their potential capacity for host shifting [6].

Unlike for HDV, for which the helper hepatitis B virus [3,18] responsible for its transmission is well described, helper viruses for most animal-associated deltaviruses remain largely unknown and most insights into potential helper viruses rely primarily on RNA-seq evidence of viral co-infections in deltavirus-positive hosts [6,9,12, 19, 21, 22]. Intriguingly, no evidence of co-infection with potential DNA or RNA helper viruses was found in any of the five runs of *S. crassicaudata* that contained FtDDeV sequences, nor in the datasets where the first marsupial deltavirus fragments were identified [23]. This could be due to several non-mutually exclusive reasons: (1) a technical inability to detect the co-infecting virus stemming from sequencing library preparation or computational search; (2) while helper viruses permitted deltaviruses to infect studied animals, they might have been cleared by the host immune system, whereas deltaviruses persisted; (3) deltaviruses might exploit other transmission strategies in marsupials that do not require a helper virus.

Interestingly, fat-tailed dunnarts have been associated with endogenous viral elements (EVEs), highlighting past infection events involving members of the *Bornaviridae* and *Filoviridae* families [43]. The presence of these EVEs indicates that fat-tailed dunnarts have also been exposed to enveloped viruses in the past and may be infected by them. It is, therefore, tempting to speculate that some of these viruses, or related ones, could theoretically provide glycoproteins required for deltavirus assembly and infectious particle production. In this regard, a recent report suggests that a newly described deltavirus in canary birds (*Serinus canaria*) might use an avian bornavirus as a helper [10]. Further investigation of the fat-tailed dunnart virome is needed to improve our understanding of potential helper viruses that may facilitate FtDDeV transmission, in particular, and deltavirus spread in general.

Together, our findings extend the confirmed host range of deltaviruses to marsupials and deliver a replication-competent clone to investigate helper usage, host restriction and deltavirus evolution, putting marsupials squarely on the deltavirus map.

## Supplementary material

10.1099/jgv.0.002203 Supplementary Material 1.

## Abbreviations

DAg: delta antigen
EVEs: endogenous viral elements
FtDDeV: fat-tailed dunnart deltavirus
HBV: hepatitis B virus
HDAg: hepatitis delta antigen
HDV: hepatitis D virus
RDeV: rodent deltavirus
SDeV: snake deltavirus
