# Supplementary material for: A replication-competent deltavirus from the marsupial fat-tailed dunnart Sminthopsis crassicaudata
Source: J Gen Virol. 2026 Jan 16;107(1):002203. doi: 10.1099/jgv.0.002203 (PMC12811629; doi:10.1099/jgv.0.002203)
Supplement: Supplementary Material 1. [file jgv-107-02203-s001.pdf]

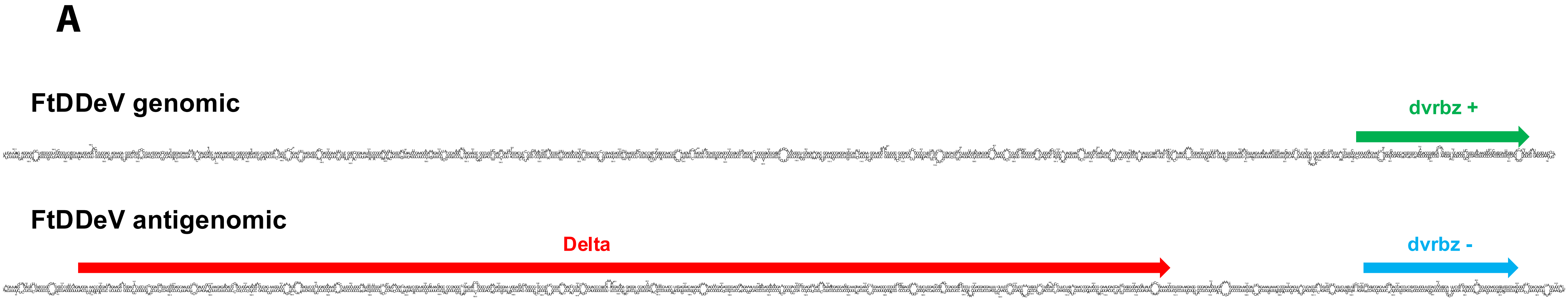

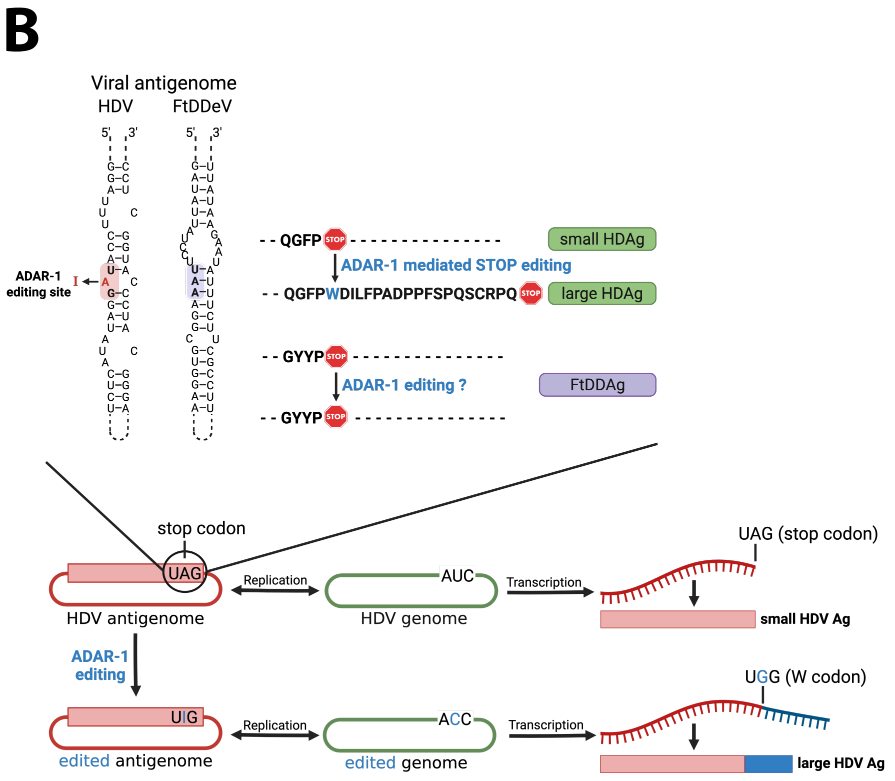

Supplementary Figure 1: (A) Secondary structure predictions of the genomic and antigenomic sequences of FtDDeV. Delta ribozymes and delta antigen ORF are indicated with arrows. **(B) ADAR1-mediated HDV antigenome editing mechanism.** ADAR-1 catalyzes an adenosine to inosine editing event at the stop-codon (UAG) on the antigenomic HDV RNA. After replication and subsequent transcription, the original stop-codon AUG is converted into UGG, coding for a tryptophan residue (W). Loss of the original stop-codon allows extension of the open reading frame translation resulting in the production of a large HDAg containing 19 additional amino acids. Antigenomic RNA secondary structure predictions of HDV and FtDDeV in the region encoding the delta antigen stop codon. The enlarged region shows the HDV amber stop-codon (left), with the ADAR-1 editable adenosine highlighted in red and the FtDDeV ochre stop-codon in purple (right).

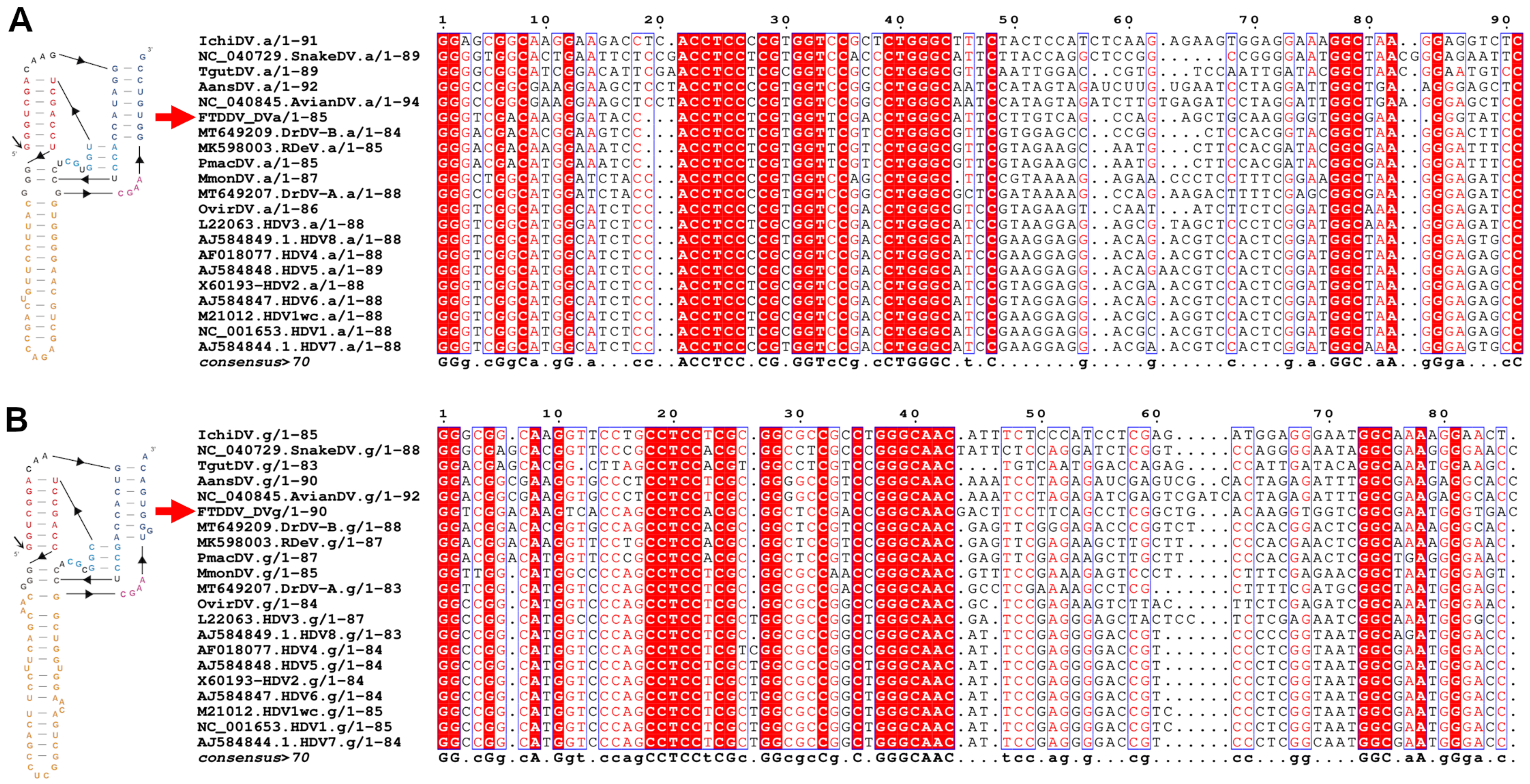

Supplementary Figure 2: Sequence alignments of the (A) genomic and (B) antigenomic delta ribozymes present in delta viruses from diverse vertebrates. The secondary structure of the delta ribozymes detected in the FtDDeV genome are depicted.

Supplementary Table 1. Presence of deltavirus sequences in RNA-seq from 2 marsupials

Host	BioProject	Run	Tissue	DV contig (nt)	Reference
Sminthopsis crassicaudata	PRJNA1028148	SRR26386756	endometrium	1680 (full circular)	This work
Sminthopsis crassicaudata	PRJNA1028148	SRR26386769	eye	266 (partial)	This work
Sminthopsis crassicaudata	PRJNA1028148	SRR26386772	prostate gland	587 (partial)	This work
Sminthopsis crassicaudata	PRJNA1028148	SRR26386778	endometrium	1378 (partial)	This work
Sarcophilus harrisii	PRJNA693818	SRR13765777	Tasmanian devil facial tumour	741 (partial)	Harvey et al. 2023
Sminthopsis crassicaudata	PRJNA554238	SRR9673767	eye	642 (partial)	Harvey et al. 2023
